# Silicon Stimulates Plant Growth and Metabolism in Rice Plants under Conventional and Osmotic Stress Conditions

**DOI:** 10.3390/plants10040777

**Published:** 2021-04-15

**Authors:** Sara Monzerrat Ramírez-Olvera, Libia Iris Trejo-Téllez, Fernando Carlos Gómez-Merino, Lucero del Mar Ruíz-Posadas, Ernesto Gabriel Alcántar-González, Crescenciano Saucedo-Veloz

**Affiliations:** 1 Department of Plant Physiology, College of Postgraduates in Agricultural Sciences Campus Montecillo, Montecillo 56230, Mexico; ramirez.sara@colpos.mx; 2 Department of Soil Science, College of Postgraduates in Agricultural Sciences Campus Montecillo, Montecillo 56230, Mexico; alcantar@colpos.mx; 3 Department of Biotechnology, College of Postgraduates in Agricultural Sciences Campus Córdoba, Amatlán de los Reyes, Veracruz 94953, Mexico; 4 Department of Botany, College of Postgraduates in Agricultural Sciences Campus Montecillo, Montecillo 56230, Mexico; lucpo@colpos.mx; 5 Department of Fruit Growing, College of Postgraduates in Agricultural Sciences Campus Montecillo, Montecillo 56230, Mexico; sauveloz@colpos.mx

**Keywords:** beneficial elements, silicon dioxide, biostimulation, *Oryza sativa* subsp. *indica*

## Abstract

Exogenous silicon (Si) can enhance plant resistance to various abiotic factors causing osmotic stress. The objective of this research was to evaluate the application of 1 and 2 mM Si to plants under normal conditions and under osmotic stress. Morelos A-98 rice seedlings, were treated with 1 and 2 mM SiO_2_ for 28 d. Subsequently, half of the plants were subjected to osmotic stress with the addition of 10% polyethylene glycol (PEG) 8000; and continued with the addition of Si (0, 1 and 2 mM SiO_2_) for both conditions. The application of Si under both conditions increased chlorophyll *b* in leaves, root volume, as well as fresh and dry biomass of roots. Interestingly, the number of tillers, shoot fresh and dry biomass, shoot water content, concentration of total chlorophyll, chlorophyll *a*/*b* ratio, and the concentration of total sugars and proline in shoot increased with the addition of Si under osmotic stress conditions. The addition of Si under normal conditions decreased the concentration of sugars in the roots, K and Mn in roots, and increased the concentration of Fe and Zn in shoots. Therefore, Si can be used as a potent inorganic biostimulant in rice Morelos A-98 since it stimulates plant growth and modulates the concentration of vital biomolecules and essential nutrients.

## 1. Introduction

Rice (*Oryza sativa*) is an extremely important crop, being the staple food of more than half of the world’s population [1,2]; however, its production requires plenty of water throughout its growing cycle, so drought stress limits its growth and yield. In addition to this, it has been reported that the frequency and severity of drought will increase in the coming years as a consequence of global climate change [3,4].

Drought has negative impacts on plant metabolism, generating morphological, biochemical, and physiological alterations that decrease production and yield [5,6]. Drought has been reported to decrease rice yield by up to 32.0%, and biomass production by 35.2% [7], in addition to decreasing plant height, number of leaves [8], and the concentrations of chlorophyll *a*, *b*, and total [9], among other changes. These challenges make it necessary to search for alternatives to stimulate plant metabolism and successfully face these stress conditions.

Silicon (Si) is a tetravalent metalloid and the most abundant element in the earth’s crust just after oxygen [10,11]. Plants can accumulate from 0.1 to 10.0% Si in their tissues [12,13]. However, the absorption mechanisms differ between species, due to the presence of carrier proteins, which is reflected in a greater accumulation of Si in some plants, such as monocots [14]. Rice is considered a Si accumulator plant because the superior ability of roots to absorb Si from the soil, as compared to other plant species [12]. This differential ability allows rice greater benefits from the application of Si in comparison to other crops [15,16].

In the range of 1 to 2.5 mM, Si has demonstrated beneficial effects not only in rice but also in durum wheat (*Triticum turgidum*) [17], sorghum (*Sorghum bicolor*) [18], cotton (*Gossypium hirsutum*) [19], tobacco (*Nicotiana tabacum*) [20], maize (*Zea mays*) [21], cucumber (*Cucumis sativus*) [22], and coffee (*Coffea arabica*) [23].

The application of Si to plants under stress conditions has shown beneficial effects in tolerance to various types of stress, both in monocotyledons and dicotyledons [24,25,26]. Under osmotic stress conditions, Si increases plant height, number of leaves, biomass weight [15,27,28], root length [7], and accumulation of osmolytes, soluble sugars, amino acids, and proline [29,30]. Si also improves the structure of thylakoids and chloroplasts, and increases the concentration of chlorophyll *a*, *b* and carotenoids [7,9]. Moreover, Si can improve the stability of the cell wall [9,15,25,31], decrease the transpiration rate [15,28], improve root hydraulic conductance and stomatal conductance, increase the expression of aquaporins and water content in the leaves [25,32], and stimulate the activity of the antioxidant enzymes superoxide dismutase, peroxidase, catalase, and glutathione reductase [15,25,26].

In Mexico, rice production is very low (i.e., 0.3 metric tons) and a major part of its demand is met by imports. Over the past 20 years, rice consumption in Mexico has grown more than 40%, with imports reaching approximately 80% of its demand. Importantly, climate change is decreasing production and yield of this grain within the country, whereas only a few studies aimed at exploring the effects of biostimulants on Mexican rice cultivars have been performed in the last decade. To date, the main rice cultivars grown in Mexico are Morelos, Milagro, and Sinaloa, which are of importance due to their cooking characteristics. Morelos is a preferred cultivar because it exhibits better culinary and industrial properties as compared to other cultivars grown within the country [33,34].

The objective of this research was to evaluate the biochemical, physiological, and nutritional responses of the Morelos A-98 rice cultivar to the application of Si supplied as silicon dioxide (SiO_2_), to plants grown under conventional conditions in the absence of stress and under stress conditions induced by polyethylene glycol (PEG).

## 2. Results

### 2.1. Si Affects the Vegetative Growth of Rice Depending on the Stress Condition

**Figure 1:**
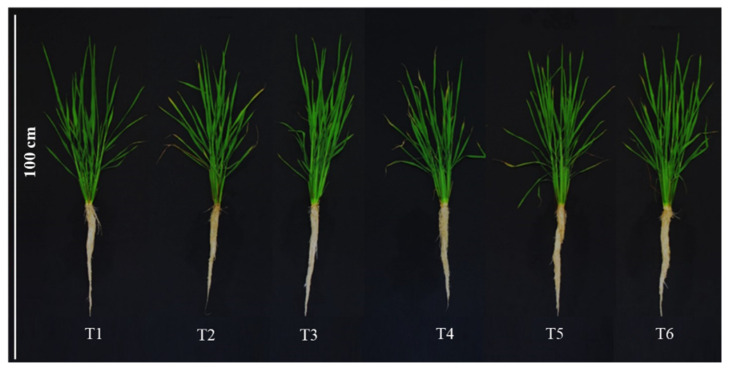
Growth of shoots and roots of rice plants treated with Si and polyethylene glycol (PEG). T1: Control; T2: 1 mM SiO_2_; T3: 2 mM SiO_2_; T4: 10% PEG 8000; T5: 10% PEG 8000 + 1 mM SiO_2_; T6: 10% PEG 8000 + 2 mM SiO_2_.

**Figure 2:**
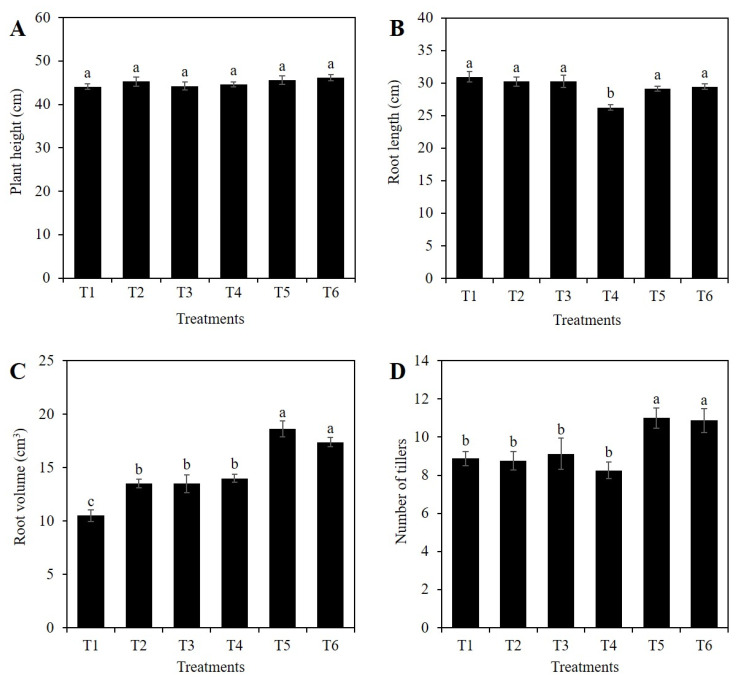
Plant height (**A**), root length (**B**), root volume (**C**) and number of tillers (**D**) of rice plants treated with Si and PEG. T1: Control; T2: 1 mM SiO_2_; T3: 2 mM SiO_2_; T4: 10% PEG 8000; T5: 10% PEG 8000 + 1 mM SiO_2_; T6: 10% PEG 8000 + 2 mM SiO_2_. The figure represents the mean of four independent replicates. Means ± SD with different letters in each variable indicate statistical differences between treatments (Duncan, *p* ≤ 0.05).

The effect of the application of Si on growth under normal conditions such as osmotic stress, is shown in Figure 1. The evaluated treatments did not affect plant height of rice (Figure 2A). Root length, however, did decrease by 15.2% in treatment T4 (10% PEG without SiO_2_), compared to the control. Furthermore, the mean value of this variable in T4 was 11.0 and 12.1% lower than the means observed in treatments T5 (1 mM Si + 10% PEG) and T6 (2 mM Si + 10% PEG), respectively (Figure 2B).

In the absence of PEG, the application of 1 mM Si (T2) and 2 mM Si (T3) increased root volume by a mean of 28.6%, compared to the control. The application of 10% PEG in the absence of Si (T4) also increased root volume by 28.6%, compared to the control. Treatments with 1 and 2 mM Si in the presence of 10% PEG (T5 and T6) increased root volume by 33 and 24.1%, compared to that recorded in plants treated only with PEG (T4), respectively (Figure 2C).

The number of tillers in the control was statistically similar to treatments T2 (1 mM Si in the absence of PEG), T3 (2 mM Si in the absence of PEG), and T4 (10% PEG in the absence of Si). In contrast, the application of 1 and 2 mM Si to plants under osmotic stress (T5 and T6) increased the number of tillers by 33.3 and 32.1%, as compared to the treatment with PEG (T4) (Figure 2D).

**Figure 3:**
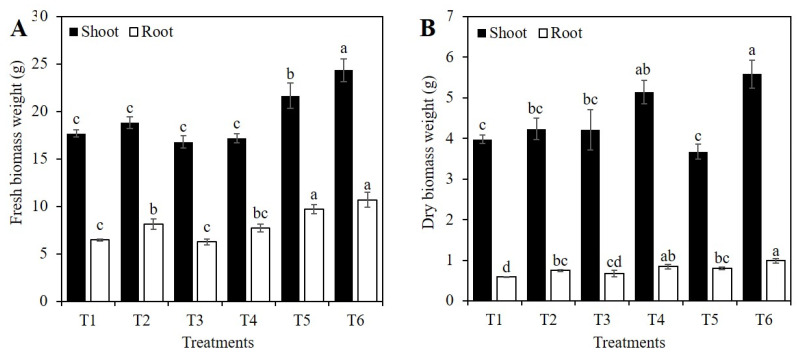
Fresh (**A**) and dry (**B**) biomass weight of shoots and roots of rice plants treated with Si and PEG. T1: Control; T2: 1 mM SiO_2_; T3: 2 mM SiO_2_; T4: 10% PEG; T5: 1 mM SiO_2_ + 10% PEG; T6: 2 mM SiO_2_ + 10% PEG. The figure represents the mean of four independent replicates. Means ± SD with different letters in each variable indicate statistical differences between treatments (Duncan, *p* ≤ 0.05).

Shoot fresh biomass weight in the control (T1) was statistically similar to treatments T2 (1 mM Si in the absence of PEG), T3 (2 mM Si in the absence of PEG), and T4 (10% PEG in the absence of Si). On the other hand, the treatments with 1 and 2 mM Si in plants subjected to PEG stress (T5 and T6) significantly increased fresh biomass weight by 26.1 and 42.8%, compared to the plants treated only with PEG (T4) (Figure 3A).

In roots, treatment T2 (1 mM Si without PEG) increased fresh biomass weight by 25.7%, compared to the control, while treatment T3 (2 mM Si without PEG) decreased the value of this variable compared to the control. Treatment T4 (10% PEG in the absence of Si) was statistically similar to the control, T2 and T3. It was interesting to note that the addition of 1 and 2 mM Si in the presence of PEG (T5 and T6) increased root fresh biomass weight by 26.1 and 38.9%, compared to plants treated only with PEG (Figure 3A).

Plants treated only with PEG (T4) surpassed the shoot dry biomass weight of the control plants by 29.4%. Likewise, plants treated with 2 mM Si and PEG (T6) surpassed the shoot dry biomass weight of the control by 41.0%. Regarding root dry weight, an increase of 27.5% was recorded in plants exposed to 1 mM Si without PEG (T2), with respect to the control. In plants exposed to 10% PEG without Si (T4), an increase of 44.7% in root dry biomass weight was observed, compared to the control. Treatments T5 (1 mM Si + 10% PEG) and T6 (2 mM Si + 10% PEG) showed no significant differences, with respect to the treatment only with PEG (T4), and these last three treatments (i.e., T4, T5, and T6) were superior to the control (T1) (Figure 3B).

**Figure 4:**
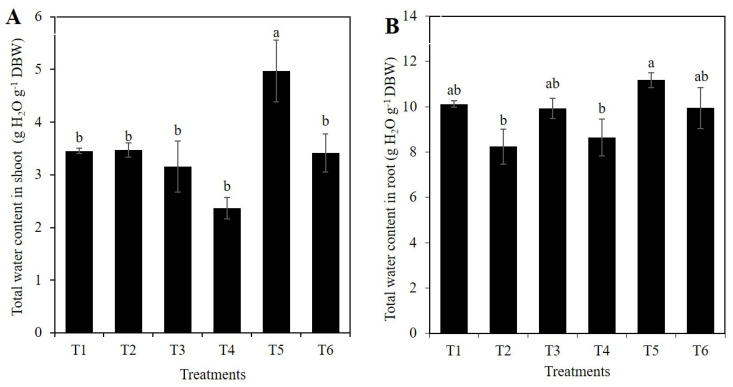
Total water content in shoot (**A**) and root (**B**) of rice plants treated with Si and PEG. T1: Control; T2: 1 mM SiO_2_; T3: 2 mM SiO_2_; T4: 10% PEG; T5: 1 mM SiO_2_ + 10% PEG; T6: 2 mM SiO_2_ + 10% PEG. The figure represents the mean of four independent replicates. Means ± SD with different letters in each variable indicate statistical differences between treatments (Duncan, *p* ≤ 0.05). DBW: Dry Biomass Weight.

Shoot water content increased by 44.0% in treatment T5 (1 mM Si + 10% PEG) compared to the control. In the rest of the treatments, no significant differences of this variable were observed with respect to the control (Figure 4A). In roots, treatment T5 (1 mM SiO_2_ + 10% PEG) was statistically superior to T2 (1 mM SiO_2_ without PEG) and T4 (10% PEG). Otherwise, the means of all the treatments were similar to the control (Figure 4B).

**Figure 5:**
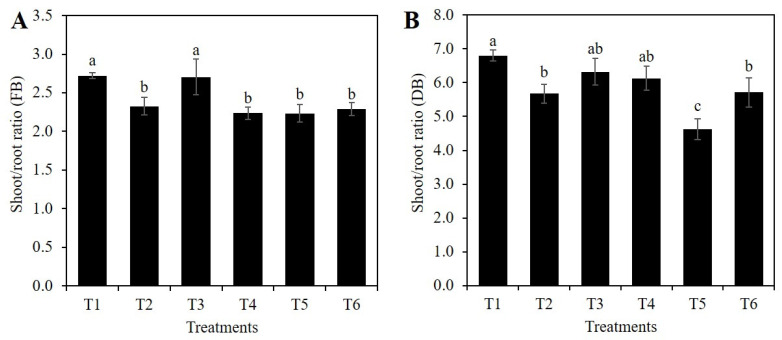
Shoot/root ratio of fresh biomass weight (**A**) and dry biomass weight (**B**) of rice plants treated with Si and PEG. T1: Control; T2: 1 mM SiO_2_; T3: 2 mM SiO_2_; T4: 10% PEG; T5: 1 mM SiO_2_ + 10% PEG; T6: 2 mM SiO_2_ + 10% PEG. The figure represents the mean of four independent replicates. Means ± SD with different letters in each variable indicate statistical differences between treatments (Duncan, *p* ≤ 0.05). FB: Fresh Biomass; DB: Dry Biomass.

The highest means of the shoot/root fresh biomass ratio were recorded in treatment T3 (2 mM Si without PEG) and the control. Means of treatments T2, T4, T5, and T6 were 14.5, 17.7, 17.1, and 15.9% lower than the control, respectively (Figure 5A). As for the shoot/root dry biomass ratio, the highest mean was observed in the control, followed by treatments T3 (2 mM Si) and T4 (10% PEG), while the lowest mean was observed in treatment T5 (1 mM SiO_2_ + 10% PEG). Treatments T2 and T5 decreased the mean value of this variable by 16.5 and 32.1%, respectively, in comparison to the control (Figure 5B).

### 2.2. Chlorophylls Concentration

Chlorophyll concentration is an important variable when monitoring the amount of solar radiation absorbed by leaves, since changes in chlorophyll concentrations will be reflected in changes of the photosynthetic process [35,36]. In addition, chlorophyll concentration is an indicator of the effects of light and water in the physiology of the plant [37]. The chlorophyll *a*/*b* ratio is related to the N partition within the leaf; when the chlorophyll *a*/*b* ratio increases, the N content in the leaf decreases [36].

**Figure 6:**
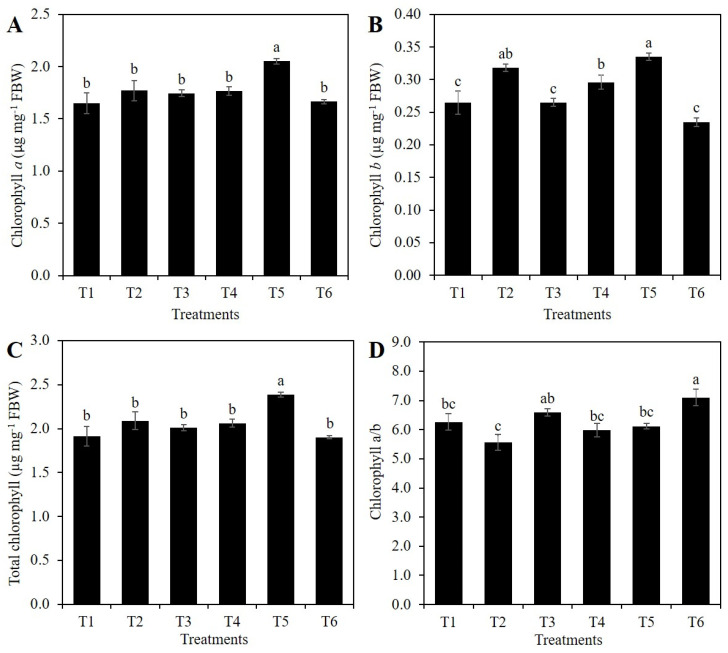
Concentration of chlorophyll *a* (**A**), *b* (**B**), total (**C**), and chlorophyll *a*/*b* ratio (**D**) of rice plants treated with Si and PEG. T1: Control; T2: 1 mM SiO_2_; T3: 2 mM SiO_2_; T4: 10% PEG; T5: 1 mM SiO_2_ + 10% PEG; T6: 2 mM SiO_2_ + 10% PEG. The figure represents the mean of four independent replicates. Means ± SD with different letters in each variable indicate statistical differences between treatments (Duncan, *p* ≤ 0.05). FBW: Fresh Biomass Weight.

The highest concentration of chlorophyll *a* was recorded in treatment T5 (1 mM Si + 10% PEG), with a mean 16.2% higher than that observed in the control. The rest of the treatments showed means statistically similar to the control (Figure 6A).

The concentration of chlorophyll *b* was the highest in treatment T5 (1 mM Si + 10% PEG), followed by treatments T2 (1 mM SiO_2_ without PEG), and T4 (only 10% PEG). The lowest values of this variable were recorded in treatments T1, T3, and T6. It is important to note that T4 (10% PEG) increased the chlorophyll *b* concentration by 11.8% compared to the control, while with the addition of SiO_2_ in T2 and T5, the mean values of this variable were 20.5 and 13.4% higher than the control (Figure 6B).

Regarding the total chlorophyll concentration, the mean observed in plants exposed to T5 (1 mM Si + 10% PEG) was 24.7% higher compared to the control. With the exception of T5, all other treatments were statistically similar to the control (Figure 6C).

Treatment T6 (2 mM Si + 10% PEG) increased the mean value of the chlorophyll *a*/*b* ratio by 18.7%, with respect to the control. Although with a lower mean value, treatment T3 was statistically similar to T6. The rest of the treatments were statistically similar to the control (Figure 6D).

### 2.3. Concentration of Free Amino Acids and Total Sugars

Osmoprotective compounds, such as amino acids, proline, and sugars, are related to cell turgor, in addition to being signaling molecules and substrates in important cellular processes under conditions of osmotic stress [38]. Furthermore, they are compounds that stabilize proteins, membranes and reduce cellular dehydration, inhibiting the effects of reactivated oxygen species as well [38,39,40].

**Figure 7:**
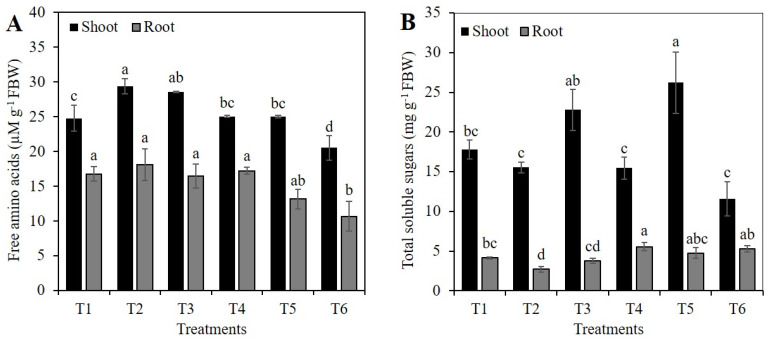
Concentration of total free amino acids (**A**) and total soluble sugars (**B**) in shoot and root of rice plants treated with Si and PEG. T1: Control; T2:1 mM SiO_2_; T3: 2 mM SiO_2_; T4: 10% PEG; T5: 1 mM SiO_2_ + 10% PEG; T6: 2 mM SiO_2_ + 10% PEG. The figure represents the mean of four independent replicates. Means ± SD with different letters in each variable indicate statistical differences between treatments (Duncan, *p* ≤ 0.05). FBW: Fresh Biomass Weight.

Treatments T2 (1 mM Si without PEG) and T3 (2 mM Si without PEG) increased the concentration of free amino acids in the shoots by 18.7 and 15.4%, respectively, compared to the control. In contrast, treatment T6 (2 mM Si + 10% PEG) decreased the amino acid concentration in the shoots by 18.1%, compared to treatment T4 (10% PEG) (Figure 7A). In roots, treatment T6 (2 mM Si + 10% PEG) caused the lowest concentration of free amino acids, followed by treatment T5 (1 mM Si + 10% PEG), although only treatment T6 was statistically different from the control; all other treatments (except T6) were statistically similar (Figure 7A).

The concentration of total sugars in the shoots of plants exposed to treatment T5 (1 mM Si + 10% PEG) was 47.4% higher than the control and 69.5% higher than treatment T4 (10% PEG), respectively. The other treatments were statistically similar to the control (Figure 7B). In roots, the highest concentration of sugars was observed in treatment T4 (10% PEG), followed by treatments T5 (1 mM Si + 10% PEG) and T6 (2 mM Si + 10% PEG). The lowest concentration of sugars was observed in T2 (1 mM Si without PEG), which was 35.0% lower than the control (Figure 7B).

### 2.4. Proline Concentration

**Figure 8:**
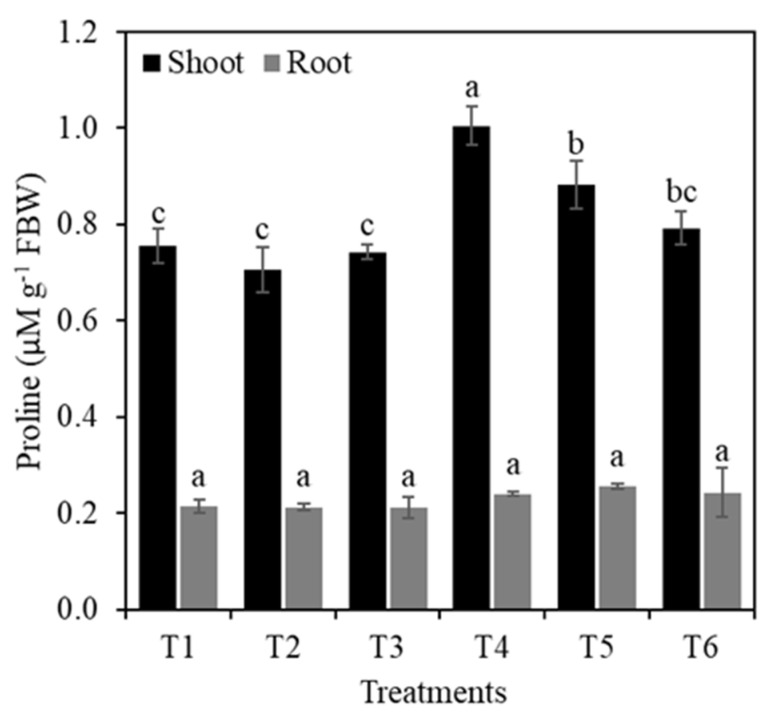
Proline concentration of rice plants treated with Si and PEG. T1: Control; T2: 1 mM SiO_2_; T3: 2 mM SiO_2_; T4: 10% PEG; T5: 1 mM SiO_2_ + 10% PEG; T6: 2 mM SiO_2_ + 10% PEG. The figure represents the mean of four independent replicates. Means ± SD with different letters in each variable indicate statistical differences between treatments (Duncan, *p* ≤ 0.05). FBW: Fresh Biomass Weight.

The highest concentration of proline was observed in treatment T4 (10% PEG), which was 33.1% higher than the control. The second highest proline value was found in treatment T5 (1 mM Si + 10% PEG). The rest of the treatments were statistically similar to the control (Figure 8). In roots, the treatments had no effect on the proline concentration (Figure 8).

### 2.5. Nutrient Concentrations

In shoots, the N, P, and Ca concentrations were influenced by the evaluated treatments. The addition of PEG (i.e., T4, T5, and T6), regardless of the Si level in the nutrient solution, significantly decreased the N concentration (by 26.2% on average), compared to the control. In contrast, treatments with only Si in the absence of PEG (T2 and T3) did not alter the concentration of N compared to the control. In general, the assayed treatments maintained similar concentrations of P in the shoot, with the exception of treatment T5 (1 mM Si + 10% PEG), which decreased the concentration of this element by 18.6%, compared to treatment T2 (1 mM Si). Likewise, the Ca concentration was similar among most of the treatments evaluated, and the lowest value was observed in treatment T3 (2 mM Si) (Table 1).

In roots, the P and Ca concentrations were not influenced by the evaluated treatments. In the case of N, a decrease of 30.1% was only observed in treatment T6 (2 mM Si + 10% PEG), compared to the control. The K concentration was significantly decreased in treatment T2 (1 mM Si without PEG), with respect to the rest of the treatments, with the exception of treatment T5 (1 mM Si + 10% PEG), with which there was no statistical difference. Furthermore, the Mg concentration in root was significantly decreased with the application of PEG, both in the absence (T4) and in the presence of Si (T5 and T6), with a mean decrease of 11.1%, compared to the control (Table 1).

In shoots, the evaluated treatments did not influence the concentrations of the micronutrients Cu, Mn, and B, but they did influence those of Fe, Zn, and Si. Treatment T2 (1 mM SiO_2_ without PEG) increased the concentrations of Fe by 36.9% and Zn by 64.6%, respectively, compared to the control (Table 2).

In roots, the treatments did not influence the concentrations of Cu and B. In all treatments, Fe concentrations were similar to the control, although numerically the highest mean was observed in T5 (1 mM SiO_2_ + 10% PEG) and the lowest in T3 (2 mM SiO_2_). The highest concentration of Mn was observed in treatment T5 (1 mM SiO_2_ + 10% PEG), and the lowest in T2 (1 mM SiO_2_) and T3 (2 mM SiO_2_). The Zn concentration showed a behavior similar to that observed in Fe, since all treatments were statistically similar to the control, with the highest mean observed in T2 (1 mM SiO_2_) and the lowest in T4 (10% PEG) (Table 2).

## 3. Discussion

Various studies have proven that the application of Si improves plant growth. For instance, in maize, the application of calcium silicate (CaOSiO_2_) improved plants height under drought conditions [28]. In tomato (*Solanum lycopersicum*) plants exposed to drought, adding sodium silicate (Na_2_SiO_3_) increased plant height by 33%, compared to control plants [41].

However, in our research, the Si treatments did not affect plant height, both with, and without, osmotic stress conditions (Figure 2A). Interestingly, when exposed to osmotic stress, Si stimulated the recovery of root length, since plants receiving either 1 mM or 2 mM Si in the presence of 10% PEG in the nutrient solution increased this variable by 12% as compared to plants exposed to 10% PEG with no Si addition (Figure 2B). Likewise, the application of Si increased root volume by up to 29% in plants without osmotic stress, compared to the control, and up to 77.4% in plants under osmotic stress (Figure 2C). Similarly, the addition of 1.5 mM potassium silicate (K_2_SiO_3_), improved the length and root volume of rice plants under osmotic stress [42]. In tomato, the application of 2.5 mM K_2_SiO_3_ in the absence of stress did not modify root length, while its application under osmotic stress conditions induced by 10% PEG-6000 increased root length by more than 50%, with no evident changes in root volume [7]. In canola (*Brassica napus*), the application of 1.7 mM Na_2_SiO_3_ modified the expression of root genes related to cell wall synthesis and degradation, as well as phytohormones enhancing root growth [43]. These findings are of utmost importance, since roots with greater length, volume, and thickness are important traits to successfully face drought events [3]. It is important to point out that the effects of Si were more marked under stress conditions. This same tendency was also observed in the number of tillers, since the application of Si without stress did not modify the number of tillers, while the application of Si to plants exposed to osmotic stress increased the number of tillers by up to 38%, compared to plants treated only with PEG (Figure 2D).

In this study, the shoot and root fresh biomass weight of plants under osmotic stress was significantly improved with the application of 2 mM Si, by up to 41.8 and 38.9%, respectively, compared to plants exposed to PEG in the absence of Si, respectively (Figure 3A). The increases in biomass weight in shoots are related to the effect of Si on photosynthesis, since most of the biomass is obtained through this process [9]. In tomato, treatment with 2.5 mM K_2_SiO_3_ under osmotic stress increased dry biomass weight by 50.6%, and also increased the expression of the proteins plastocyanin and ferredoxin, improving photosynthetic activity [7]. Si increases the activity of the photosystem II reaction center, electron transport chain, carbon fixation, and nitrogen metabolism [9,44]. The aforementioned is essential in stress conditions, since during drought there is a significant reduction in photosynthetic activity [6,9]. Nonetheless, the positive effects of Si in plants with osmotic stress were not observed in the shoot and root dry biomass in our experimental conditions (Figure 3B).

As the drought becomes harsher, the water potential of the plant decreases; however, the diurnal variation of the water potential can be decreased if transpiration is suppressed by stomatal closure, which negatively affects CO_2_ fixation [6]. Under this circumstance, the application of Si may bring benefits by decreasing transpiration [28], improving the hydraulic conductance of the roots, and increasing the expression of aquaporin proteins, as well as the K concentration in the xylem [18,45]. In addition, the potential effect of the application of Si on the absorption and distribution of K may be involved in the regulation of the water status of the plant, only under conditions of potassium deficiency [46]. By depositing in the occlusive cells of the stomata and modifying the stomatal conductance, Si can regulate water status in plants [47]. In this research, the PEG treatment decreased the water content in the shoot by 31.3%, compared to the control; on the contrary, the treatment with 1 mM Si and PEG increased the water content in shoots by 89.1%, compared to the plants treated only with PEG (Figure 4A). On the other hand, the root water content in plants under osmotic stress treated with 1 mM Si increased by 29.2%, compared to plants with only osmotic stress (Figure 4B). Thus, our findings are in full agreement with those previously described.

Under water stress conditions, an improvement in water absorption is achieved by increasing the root/shoot ratio [3]. In this research, the application of Si to plants without osmotic stress decreased the shoot/root ratio of fresh biomass by 14.5%, while in plants with osmotic stress, Si decreased this variable by up to 17.9%, compared to the control (Figure 5A). On the other hand, the shoot/root ratio of dry biomass decreased by 24.5% in plants with osmotic stress treated with 1 mM Si, compared to plants treated only with PEG (Figure 5B). This response can be attributed to a greater biomass distribution in the root with respect to the shoot, and therefore in the distribution of carbohydrates, associated with the activity of enzymes involved in the conversion of sucrose into other compounds [48]. Root growth of rice plants under osmotic stress has been reported to be positively correlated with the activity of the sucrose-phosphate synthase enzyme in shoots and the activity of the alkaline invertase enzyme in roots [49]. Coincidentally, the addition of 2 mM Si Na_2_SiO_3_ to rice plants cv. MTU1010 under salt stress conditions (25 mM NaCl) has been reported to significantly increase the activity of the sucrose-phosphate synthase enzyme in shoots [50]. Furthermore, the addition of Na_2_SiO_3_ to cucumber plants under saline stress increased the concentration of starch and sucrose in roots [51].

Chlorophyll is the most important pigment in photosynthesis, and it is severely affected under drought conditions. In plants exposed to drought stress, the application of Si has improved the stability of the thylakoid membrane and the performance of photosynthesis, while increasing antioxidant capacity and decreasing oxidative damage [7,9]. In this research, the application of 1 mM Si to plants under osmotic stress, significantly increased the concentration of chlorophyll *a* (Figure 6A), *b* (Figure 6B), and total (Figure 6C), while the addition of 2 mM Si under stress conditions significantly increased the chlorophylls *a*/*b* ratio (Figure 6D). In tomato plants exposed to osmotic stress, the application of 2.5 mM K_2_SiO_3_ significantly increased the concentration of chlorophylls *a* and *b*, as well as that of carotenoids, while the addition of Si in non-stressed plants did not modify the concentration of these molecules [7]. Rice plants exposed to osmotic stress, the application of 0.5 mM Na_2_SiO_3_ increased the concentrations of chlorophyll *a* and *b* by more than 50%, compared to plants not treated with Si [9]. Under drought stress conditions, Si can maintain the chlorophyll content and photosynthesis performance, by improving the integrity, stability, and functions of the thylakoid membrane, as well as reducing the degradation of membrane protein complexes caused by the stress imposed [9]. The loss of most thylakoid protein complexes under salinity stress are largely improved in the presence of Si [52]. In particular, Si applications have been proven to increase the *PetC* (*photosynthetic electron transport cytochrome c1*) gene’s rapid expression, protect the cytochrome in leaves and alleviate the destructive effects of high Zn on the chloroplasts’ structure. The PetC protein is strongly associated with the cytochrome bf complex on the lumenal side of the thylakoid membrane, and is involved in the cytochrome’s biological processes in photosynthesis [53].

Si can modify water transport by adjusting the osmotic potential of cells through a higher accumulation of osmolytes. Within these osmolytes are sugars and amino acids such as proline, which improve cell turgor and water absorption [3,25,28], and increase the tolerance of plants to stress conditions [54]. In this study, the concentration of total free amino acids in shoots was increased with the addition of Si only in treatments without osmotic stress. Contrarily, the 2 mM Si treatment in the presence of osmotic stress decreased the concentration of amino acids in shoots by 18.1%, compared to the treatment with PEG (Figure 7A). In roots, it was also observed that, under osmotic stress, 2 mM Si decreased the concentration of free amino acids by 73.3%, compared to plants under osmotic stress and without Si (Figure 7A).

Low sugar levels in plant tissue stimulate photosynthesis and mobilization and export of reserves [50,55]. Moreover, the sugar content in the plant is sensitive to osmotic stress, because this type of stress decreases photosynthetic efficiency, which in turn decreases the sugar content in the sink organs [38]. In this research, treatment T5 (1 mM Si + 10% PEG) caused the highest sugar concentration in shoots, while treatment T2 (1 mM Si without PEG) significantly decreased the concentration of sugars in the roots (Figure 7B). These findings demonstrate the stimulating effect of Si in improving photosynthetic activity and hence photosynthate mobilization [42].

Proline has an extremely important role during drought stress, since it is an antioxidant molecule and participates in cell signaling. Under stress conditions, proline increases the antioxidant capacity of plants [3]. In this research, the concentration of proline in shoots was not modified with the addition of Si to plants without osmotic stress, while under osmotic stress, increases in proline were observed; however, they were negatively related to the Si concentration supplied. In drought conditions, Si can improve transpiration, which would drive more water from the root to the shoot, reflecting the increase in the osmotic potential and reduction of proline concentrations [15]. In treatment T4 (10% PEG), proline in the shoots increased by 33.1% compared to the control; in treatment T6 (2 mM Si + 10% PEG), it increased by 4.1% but such increase was not statistically different from the control (Figure 8).

Drought generates a negative impact on water metabolism, affecting the transport, accessibility, and absorption of essential nutrients by modifying their absorption, mobilization, and distribution within the cells and tissues, which interferes with physiological and biochemical processes, thus reducing growth, development, and yield [6,9,54]. In our study, treatment with PEG decreased N concentration in shoots and Mg concentration in roots. Drought decreases the activity of enzymes involved in N metabolism, such as the nitrate reductase enzyme [46], as well as the expression of high affinity nitrate transporter genes such as *NAR2.1*, *NRT2.1*, and *NRT2.2* in leaves and roots of rice plants [56]. Furthermore, during drought, the accumulation of N in roots is stimulated and the absorption of N decreases, which generates a decrease in the concentration of N in leaves [57]. Likewise, under drought stress conditions, the transport of Mg to the aerial part is favored as a strategy to avoid a decrease in photosynthetic activity [58].

Si can modify nutritional status, which may be due to its effect on transpiration, root hydraulic conductance, stomatal conductance, and water content [10,12,32,59]. Furthermore, Si is involved in essential nutrient homeostasis under stress conditions by regulating nutrient deficiency or toxicity [60,61]. In the present research, the concentrations of K and Ca in shoots and P and Ca in roots were not modified with the evaluated treatments (Table 1). Likewise, in sorghum, Si application did not influence the K^+^ concentration in leaves under K-sufficient or K-deficient conditions, suggesting that Si’s ability to alleviate K-deficiency-induced leaf chlorosis is not due to increased leaf K^+^ concentration [18].

In barley (*Hordeum vulgare*), grown under osmotic stress induced by PEG, the application of Si did not affect the P concentration under the evaluated conditions [62], which is consistent with our study. Contrastingly, the addition of 1 mM H_4_SiO_4_ to rice plants significantly decreased the concentration of P in shoots and roots, as well as the absorption rate of P, and decreased the expression of the *OsPT6* phosphate transporter [59].

Regarding the concentration of micronutrients (Table 2), the addition of 1 mM Si significantly increased the Fe concentration in shoots in the absence of osmotic stress, without showing effects on the concentration in roots. This may be due to the effect of Si on the acquisition of Fe from the roots towards the aerial part and an increase in the apoplastic reserve of Fe in roots [22]. Coincidentally, the addition of Si to field salad plants (*Valerianella locusta*) under conditions of Fe sufficiency improved Fe acquisition by the roots [63]. Likewise, in the absence of osmotic stress, the addition of 1 mM Si decreased the concentration of Mn in roots, while in conditions of osmotic stress the concentration of this element increased (Table 2). Further, in rice, the addition of 1 mM Si from H_4_SiO_4_ significantly decreased the concentration of Mn in shoots, without showing significant effects in roots, which may be due to the effect of Si on the regulation of the *OsNramp5* gene, as well as on the formation of Mn-Si complexes in the root cytosol [64]. With respect to B, the lowest concentration in shoots was obtained with treatment T2 (1 mM Si in the absence of PEG), without showing significant effects on roots (Table 2). In oats (*Avena sativa*), the application of Si decreased the concentration of B [65]. In this research, the Zn concentration in shoots increased with the application of 1 mM in the absence of stress, without showing root effects (Table 2). Similar results were obtained with Si application during the flowering stage in strawberry (*Fragaria* × *ananassa*) plants where the Zn concentration increased by 30.2% [44].

From our data, we can observe that roots contain more Fe, B, and Zn as compared to shoots. In plants, essential nutrients move from roots to stems, from stems to leaves, and from leaves to fruits and seeds. Nevertheless, within the plant, Fe, B, and Zn are less mobile as compared to other essential elements such as N, P, and K [66]. In rice plants grown under iron toxicity, tolerant cultivars may store more Fe in its shoots and less in its leaves than do the sensitive cultivars [67,68,69]. Indeed, in most rice genotypes, only a few Fe, B, and Zn acquired by roots is translocated to shoots [70].

## 4. Materials and Methods

### 4.1. Plant Material, Seed Disinfection

We used rice (*Oryza sativa* L. subsp. *indica*) seeds of the cultivar Morelos A-98 obtained from the National Rice Germplasm Bank of the National Institute of Forestry, Agriculture and Livestock Research (INIFAP), located in the Zacatepec Experimental Field, Morelos, Mexico (18°39′ NL, 99°12′ WL, 910 masl). Seeds were disinfected with 70% alcohol for 10 min, then immediately rinsed with sterile distilled water and immersed for 1 h in a mixture of 5% sodium hypochlorite and 0.1% Tween™ 20 (Hycel; Zapopan, Mexico).

### 4.2. Vegetative Growth

The disinfected seeds were placed in glass jars with 60 mL MS medium (Sigma-Aldrich; Steinheim, Germany) supplemented with 3% (*w*/*v*) sucrose (J. T. Baker; Center Valley, PA, USA), and solidified with 0.8% agar (Merck; Darmstadt, Germany). The jars were then placed in the dark at 28 °C for 3 d, followed by a period of 11 d exposed to natural light at room temperature. Once seedlings reached 12 d of age, they were transferred to a hydroponic system under greenhouse conditions at a mean temperature of 23 °C/16 °C (day/night), relative humidity of 57%, with 16 h light at 145 μmol m^−2^ s^−1^ and 8 h darkness. Plants were placed in 14 L containers with Magnavaca nutrient solution [71] modified by Famoso et al. [72], made up of 1 mM KCl, 1.5 mM NH_4_NO_3_, 1 mM CaCl_2_ 2H_2_O, 45 µM KH_2_PO_4_, 200 µM MgSO_4_ 7H_2_O, 500 µM Mg(NO_3_)_2_ 6H_2_O, 155 µM MgCl_2_ 6H_2_O, 11.8 mM MnCl_2_ 4H_2_O, 33 µM H_3_BO_3_, 3 µM ZnSO_4_ 7H_2_O, 0.8 µM CuSO_4_ 5H_2_O, 1 µM NaMoO_4_ 2H_2_O, and 77 µM Fe-EDTA. Seven days after transplanting, the Magnavaca solution was replaced by the Yoshida solution [73], consisting of 1.43 mM NH_4_NO_3_, 1.00 mM CaCl_2_ 2H_2_O, 1.64 mM MgSO_4_ 7H_2_O, 1.32 mM K_2_SO_4_, 320 µM NaH_2_PO_4_, 100 µM Fe-EDTA, 7.99 µM MnCl_2_ 4H_2_O, 0.15 µM ZnSO_4_ 7H_2_O, 0.15 µM CuSO_4_ 5H_2_O, 0.08 µM (NH_4_)_6_Mo_7_O_24_ 4H_2_O, and 1.39 µM H_3_BO_3_. Fourteen days after transplanting (26 d old plants), Si treatments (0, 1, 2, mM SiO_2_) were applied through the nutrient solution. Twenty-eight days after the start of the Si treatments, half of the plants were subjected to osmotic stress for 7 days with the addition of 10% PEG 8000 (PEG), maintaining the described SiO_2_ levels. The rest of the plants continued to be exposed to SiO_2_ levels also for 7 d. After the addition of the SiO_2_ treatments, the nutrient solution was completely replaced every seven days, while the water consumed by the plant was replenished every other day. The pH of the solution was adjusted to 5.5 using H_2_SO_4_ or 1 N NaOH. The osmotic potentials of the six evaluated treatments were determined with a Vapro 5520 osmometer (Wescor; Logan. UT, USA). The treatments and their osmotic potentials, shown in parentheses, were: T1: Control (−0.09 MPa), T2: 1 mM SiO_2_ (−0.09 MPa), T3: 2 mM SiO_2_ (−0.09 MPa), T4: 10% PEG (−0.180 MPa), T5: 1 mM SiO_2_ + 10% PEG (−0.198 MPa), and T6: 2 mM SiO_2_ + 10% PEG (−0.183 MPa).

After 35 d of treatments with SiO_2_ and 7 d with and without PEG induced stress treatment, the 60 d old plants were removed from the nutrient solution and rinsed; plant height, root length and volume, number of tillers, and fresh biomass weight were recorded. The root volume was obtained by displacement, for which each root was placed in a graduated cylinder, which contained water, and the difference between the initial volume and the volume displaced by the root, the volume of the root was considered. The plants were separated into shoot and root, dried in a forced air oven (HCF-125; Riossa, Mexico) at 72 °C for 72 h, and the dry biomass weight was determined. Subsequently, with the fresh and dry biomass weight data, the shoot/root ratio was calculated and the total shoot and root water content was calculated according to Jones and Turner [74] and Ming et al. [75], with the following formula:

$$\text{Total}\;\text{water}\;\text{content}=\frac{\left(\text{Fresh}\;\text{weight}-\text{Dry}\;\text{weight}\right)}{\text{Dry}\;\text{weight}\;}$$

### 4.3. Chlorophylls Concentration

Shoot and root fresh tissue samples were separately macerated in liquid nitrogen. Subsequently, 60 mg of each sample were weighed, and a triple ethanol extraction was performed (80, 80, and 50%). In each extraction, the samples were incubated in a water bath at 80 °C for 20 min and centrifuged at 14,000 rpm for 5 min. The supernatants from each extraction were recovered, mixed, and used for the determination of chlorophylls and amino acids. Finally, the concentrations of chlorophylls *a* and *b* in shoots were determined by reading the extracts at 635 and 645 nm in a Jenway 6715 spectrophotometer (Cole-Parmer; Staffordshire, UK), and the total chlorophyll concentration was calculated, as well as the chlorophyll *a*/*b* ratio [76].

### 4.4. Concentrations of Amino Acids and Total Sugars

From the supernatant obtained in the chlorophyll determination, the total free amino acid concentration was determined in shoots and roots using the ninhydrin method described by Moore and Stein [77], and the samples were analyzed at 570 nm. L-leucine (Sigma-Aldrich; Steinheim, Germany) was used for the preparation of the standard curve.

Total sugars were determined in shoots and roots. To do this, 500 mg of fresh tissue, previously macerated in liquid nitrogen, were weighed. Subsequently, the extraction was carried out with 50 mL ethanol (80%) on a hot plate at constant boiling with occasional stirring. The obtained supernatant was filtered and measured to 20 mL, from which 1 mL of the extract was taken and 5 mL of 0.4% anthrone (*w*/*v*) was added in concentrated sulfuric acid (Merck; Darmstadt, Germany). Then, the samples were incubated in a water bath at 95 °C for 15 min. After this, the samples were placed on ice to finalize the reaction. The quantification was performed on a standard curve using glucose (Sigma-Aldrich; Saint Louis, MO, USA) and measured in the spectrophotometer at an absorbance of 600 nm [78].

### 4.5. Proline Concentration

To determine proline concentration in plant tissues, 0.5 g shoot and 1 g root were macerated with sulfosalicylic acid (3%, *w*/*v*) and the sample was filtered (filter paper No. 4). Then, 2 mL of the obtained extract was mixed with 2 mL acid ninhydrin and 2 mL concentrated glacial acetic acid. Subsequently, it was incubated in a water bath at 100 °C for 60 min. Immediately thereafter, samples were placed on ice and 4 mL toluene (J. T. Baker) were added and the mixture was stirred; absorbance was recorded at 520 nm. The proline concentration was determined through a standard curve using L-Proline [79].

### 4.6. Nutrient Analysis

Shoot and root dry tissues were separately ground and run through a 40 mesh screen, then 0.25 g of each tissue were weighed and subjected to a wet digestion with a mixture of H_2_SO_4_ and HClO_4_ (2/1, *v*/*v*). At the end of the digestion, the extracts obtained were filtered and measured to 25 mL with de-ionized water. Subsequently, the extracts were read in an inductively coupled plasma optical emission spectrometer (Varian ICP OES 725-ES; Mulgrave, Australia) to determine the elements, except for N, which was determined by the micro Kjeldahl method [80].

### 4.7. Statistical Analysis

Treatments tested were distributed in a completely randomized experimental design. Data obtained were subject to an analysis of variance and means were compared using the Duncan test with α = 0.05. The SAS version 9.3 statistical software (SAS Institute; Cary, NC, USA) was used to perform all statistical analyses.

## 5. Conclusions

The addition of Si in the absence and presence of PEG modified growth and the concentration of biomolecules and nutrients in rice plants. In the absence of PEG in the nutrient solution, the addition of Si increased root volume, root fresh and dry biomass, and the concentration of chlorophyll *b* in shoots. In the presence of PEG, Si significantly increased root length and volume, number of tillers, root and shoot fresh and dry biomass, total water content in shoots and roots, chlorophyll *a*, *b* and total, as well as the chlorophyll *a*/*b* ratio. Concentrations of total soluble sugars and proline were also increased with the addition of Si in the presence of PEG. Nutrient concentrations were differentially affected by the treatment tested. In shoots, N, P, Ca, Fe, B, and Zn were modified, whereas in roots the effects of the treatments were observed on N, K, Mg, Fe, Mn, and Zn. In summary, Si showed stimulating effects on the growth and metabolism of rice plants, both under stress-free conditions and in the presence of osmotic stress induced by PEG. Therefore, the supply of Si to rice plants can enhance growth and improve metabolism, while increasing tolerance to osmotic stress imposed by PEG, and reducing water consumption. These findings are of paramount importance for countries like Mexico that are facing environmental challenges imposed by global warming and climate change.

## Figures and Tables

**Table 1:** Macronutrient concentration in shoots and roots of rice plants treated with Si and PEG.

TRE	Macronutrient Concentration (g kg^−1^ Dry Biomass Weight)				
	N	P	K	Ca	Mg
	Shoots				
T1	35.96 ± 0.92 a	5.46 ± 0.27 ab	21.10 ± 0.65 a	1.36 ± 0.02 ab	3.45 ± 0.11 a
T2	36.75 ± 1.29 a	6.02 ± 0.09 a	22.38 ± 0.93 a	1.52 ± 0.08 ab	3.66 ± 0.10 a
T3	38.59 ± 0.99 a	5.45 ± 0.39 ab	21.47 ± 0.85 a	1.32 ± 0.06 b	3.17 ± 0.21 a
T4	27.48 ± 1.79 b	5.67 ± 0.34 ab	24.05 ± 2.11 a	1.55 ± 0.07 a	3.48 ± 0.27 a
T5	23.45 ± 3.99 b	4.90 ± 0.14 b	22.97 ± 0.95 a	1.58 ± 0.09 a	3.16 ± 0.10 a
T6	28.70 ± 1.59 b	5.31 ± 0.21 ab	24.29 ± 0.92 a	1.53 ± 0.07 ab	3.39 ± 0.14 a
	Roots				
T1	20.04 ± 0.92 a	2.39 ± 0.11 a	17.71 ± 0.53 a	0.97 ± 0.03 a	1.41 ± 0.03 a
T2	16.45 ± 1.36 ab	2.52 ± 0.09 a	15.20 ± 0.95 b	0.96 ± 0.03 a	1.35 ± 0.03 ab
T3	18.90 ± 1.64 ab	2.58 ± 0.09 a	17.22 ± 0.46 a	0.92 ± 0.03 a	1.42 ± 0.04 a
T4	18.03 ± 2.67 ab	2.45 ± 0.13 a	18.34 ± 0.53 a	1.03 ± 0.09 a	1.22 ± 0.02 c
T5	16.63 ± 1.16 ab	2.50 ± 0.09 a	16.57 ± 0.40 ab	1.02 ± 0.02 a	1.28 ± 0.01 bc
T6	14.00 ± 1.48 b	2.41 ± 0.13 a	17.91 ± 0.69 a	1.05 ± 0.04 a	1.26 ± 0.02 bc

TRE: Treatments. T1: Control; T2: 1 mM SiO_2_; T3: 2 mM SiO_2_; T4: 10% PEG; T5: 1 mM SiO_2_ + 10% PEG; T6: 2 mM SiO_2_ + 10% PEG. The table represents the mean of four independent replicates. Means ± SD with different letters in each variable indicate statistical differences between treatments (Duncan, *p* ≤ 0.05).

**Table 2:** Concentration of micronutrients in shoots and roots of rice plants treated with Si and PEG.

TRE	Micronutrient Concentration (mg kg^−1^ Dry Biomass Weight)				
	Fe	Cu	Mn	B	Zn
Shoots					
T1	162.8 ± 13.5 b	3.4 ± 0.1 a	179.8 ± 8.6 a	12.5 ± 1.0 ab	13.7 ± 0.7 b
T2	222.8 ± 11.1 a	3.6 ± 0.2 a	200.2 ± 8.0 a	9.4 ± 0.7 b	22.5 ± 1.3 a
T3	153.4 ± 15.9 b	4.1 ± 0.5 a	179.0 ± 25.2 a	11.5 ± 1.1 ab	12.2 ± 2.6 b
T4	136.5 ± 6.6 b	3.2 ± 0.3 a	219.2 ± 30.0 a	13.4 ± 1.4 ab	13.5 ± 1.7 b
T5	142.0 ± 7.9 b	3.0 ± 0.1 a	210.8 ± 18.2 a	14.0 ± 2.2 a	15.6 ± 2.4 b
T6	133.2 ± 4.1 b	3.7 ± 0.7 a	233 ± 26.4 a	9.4 ± 0.9 b	13.4 ± 1.7 b
Roots					
T1	717.6 ± 248.0 ab	3.7 ± 0.5 a	34.3 ± 2.3 c	34.3 ± 3.8 a	28.7 ± 1.5 ab
T2	1044.5 ± 206.3 ab	4.3 ± 0.5 a	25.4 ± 0.8 d	33.6 ± 2.2 a	29.7 ± 2.2 a
T3	570.3 ± 150.6 b	3.8 ± 0.4 a	28.4 ± 0.7 cd	32.7 ± 2.8 a	27.6 ± 1.2 ab
T4	909.7 ± 189.4 ab	3.7 ± 0.4 a	46.0 ± 3.5 b	35.0 ± 4.2 a	22.7 ± 0.9 b
T5	1533.6 ± 427.8 a	4.0 ± 0.4 a	53.8 ± 2.6 a	30.1 ± 3.7 a	27.3 ± 2.1 ab
T6	1089.7 ± 395.4 ab	4.4 ± 0.4 a	43.7 ± 2.5 b	26.4 ± 1.5 a	27.9 ± 2.9 ab

TRE: Treatments. T1: Control; T2: 1 mM SiO_2_; T3: 2 mM SiO_2_; T4: 10% PEG; T5: 1 mM SiO_2_ + 10% PEG; T6: 2 mM SiO_2_ + 10% PEG. The table represents the mean of four independent replicates. Means ± SD with different letters in each variable indicate statistical differences between treatments (Duncan, *p* ≤ 0.05).

## Data Availability

The available data are presented in the manuscript.
